# Effects of Spinal Cord Stimulation in Patients with Small Fiber and Associated Comorbidities from Neuropathy After Multiple Etiologies

**DOI:** 10.3390/jcm14020652

**Published:** 2025-01-20

**Authors:** Ángeles Canós-Verdecho, Ara Bermejo, Beatriz Castel, Rosa Izquierdo, Ruth Robledo, Elisa Gallach, Teresa Sevilla, Pilar Argente, Ismael Huertas, Isabel Peraita-Costa, María Morales-Suarez-Varela

**Affiliations:** 1 Multidisciplinary Pain Management Unit, La Fe University and Polytechnic Hospital, Av. de Fernando Abril Martorell, 106, 46026 Valencia, Spain; canos_marver@gva.es (Á.C.-V.); arabermejo@hotmail.com (A.B.); beatrizcastle@gmail.com (B.C.); rosizaguirre@hotmail.es (R.I.); robledo_rut@gva.es (R.R.); gallach.eli@gmail.com (E.G.); 2 Anaesthesiology Department, La Fe University and Polytechnic Hospital, Av. de Fernando Abril Martorell, 106, 46026 Valencia, Spain; argente_marnav@gva.es; 3 Neurology Department, La Fe University and Polytechnic Hospital, Av. de Fernando Abril Martorell, 106, 46026 Valencia, Spain; sevilla_ter@gva.es; 4 Psychiatry Department, La Fe University and Polytechnic Hospital, Av. de Fernando Abril Martorell, 106, 46026 Valencia, Spain; 5 CIBER of Rare Diseases (CIBERER), Carlos III Health Institute (ISCIII), Av. Monforte de Lemos, 3-5, Pabellón 11, Planta 0, 28029 Madrid, Spain; 6 Department of Medicine, Faculty of Medicine, Universitat de València, Av. Blasco Ibáñez 15, 46010 Valencia, Spain; 7 Neuromuscular and Ataxias Research Group, Instituto de Investigación Sanitaria La Fe, Av. de Fernando Abril Martorell, 106, 46026 Valencia, Spain; 8 Boston Scientific Neuromodulation Research (NRAC), 25155 Rye Canyon Loop, Valencia, CA 91355, USA; ismhuefer@gmail.com; 9 Research Group in Social and Nutritional Epidemiology, Pharmacoepidemiology and Public Health, Department of Preventive Medicine and Public Health, Food Sciences, Toxicology and Forensic Medicine, Faculty of Pharmacy, Universitat de València, Av. Vicent Andrés Estellés s/n, 46100 Burjassot, Spain; isabel.peraita@uv.es; 10 CIBER of Epidemiology and Public Health (CIBERESP), Carlos III Health Institute (ISCIII), Av. Monforte de Lemos, 3-5, Pabellón 11, Planta 0, 28029 Madrid, Spain

**Keywords:** small fiber neuropathy, spinal cord stimulation, peripheral polyneuropathy, sub-perception SCS, personalized neuromodulation

## Abstract

**Objectives:** The aim of this study was to evaluate the effects of spinal cord stimulation (SCS) on pain, neuropathic symptoms, and other health-related metrics in patients with chronic painful peripheral neuropathy (PN) from multiple etiologies. **Methods:** A prospective single center observational longitudinal cohort study assessed SCS efficacy from April 2023 to May 2024, with follow-ups at 2, 4, 6, and 12 months in 19 patients suffering from the painful polyneuropathy of diverse etiologies: diabetic (DPN), idiopathic (CIAP), chemotherapy-induced (CIPN), and others. Patients were implanted with a neurostimulator (WaveWriter Alpha^TM^, Boston Scientific Corporation, Valencia, CA, USA) and percutaneous leads targeting the lower limbs (T10–T11) and, if necessary, the upper limbs (C4–C7). Stimulation programming was individualized based on patient preference and best response. Assessments were performed before and after implantation and included pain intensity (VAS and DN4), neuropathic pain symptoms (NPSI and SF-MPQ-2), autonomic symptoms (SFN-SIQ and SAS), sensory and small fiber nerve injury (UENS), functionality (GAF), sleep (CPSI), global impression of change (CGI and PGI), and quality of life (EQ-VAS and EQ-5D). Intra-epidermal nerve fiber density (IENFD) via skin biopsy was also performed at baseline (diagnostic) and after 12 months to assess potential small fiber re-growth. Statistical analyses were conducted to determine the evolution of treatment success. **Results:** To date, 19 patients have undergone implantation and completed follow-up. SCS produced a significant consistent and sustained improvement in pain intensity by 49% in DN4 and 76% in VAS, in neuropathic pain symptoms by 73%, in autonomic symptoms by 26–30%, in the sensorimotor physical exam by 8%, in functionality by 44%, in sleep by 74%, and in quality of life (69% for EQ-VAS and 134% EQ-5D). Both clinicians and patients had a meaningful global impression of change, at 1.1 and 1.3, respectively. Distal intra-epidermal nerve fiber density improved by 22% at 12 months while proximal intra-epidermal nerve fiber density decreased by 18%. **Conclusions:** SCS is an effective therapy for managing various types of PN.

## 1. Introduction

Chronic polyneuropathy (PN) is a type of peripheral neuropathy characterized by a generalized disease of the peripheral nerves that typically causes symmetrical sensory symptoms, predominantly in the extremities, such as numbness, paresthesia, muscle weakness, and ultimately pain [1]. Over 100 potential causes and risk factors for chronic PN have been identified [2]. However, in one quarter to a third of cases, the origin or cause cannot be established [3], and it is termed chronic idiopathic axonal polyneuropathy (CIAP) [4]. It is estimated that millions of people suffer from PN worldwide [5,6].

Pain and neuropathic symptoms, such as those suffered by people with PN, can be debilitating and can severely interfere with daily activities and quality of life. The pain is often a burning sensation and may be worse at night, thus interfering with sleep. Allodynia and cramps may also occur. Autonomic dysfunction can affect multiple organs and systems and cause symptoms such as sweating abnormalities, orthostatic intolerance, and gastrointestinal and urogenital symptoms, among other things [7,8,9].

Conventional treatment consists of pharmacological agents such as gabapentinoids (i.e., gabapentin, pregabalin), antidepressants (i.e., duloxetine, amitriptyline), or opioids [10]. Unfortunately, the effectiveness of these drugs is often not profound or durable over time, so that patients continue to suffer in the long-term. It has been estimated that up to 50% of diabetic PN (DPN) patients discontinued these medications within 3 months of initiation [11]. In these cases, spinal cord stimulation (SCS) offers an interesting solution [12].

Tesfaye et al. already showed in 1996 that SCS was effective for treating a cohort of patients with painful, drug-resistant DPN [13]. More recently, three RCTs showed that SCS was superior to conventional medical management for DPN [14,15,16]. Recent systematic reviews of neurostimulation for painful diabetic neuropathy confirmed the benefits of SCS for patients with DPN [17,18,19]. Also, other case series have reported effective results to treat other etiologies such as chemotherapy-induced PN (CIPN) [20] or HIV-induced PN [21]. However, despite this evidence, these patients are not routinely referred to pain interventionalists, and global clinical experience of SCS is still limited, as is the existing literature. For example, the effectiveness of SCS to treat other PN subtypes such as CIAP, accounting for up to a third of the global cases [22], is unknown.

In this work, the aim was to investigate the effectiveness of SCS, quantified by the improvement in the selected outcome measures, to treat painful chronic PN of various etiologies.

## 2. Materials and Methods

The study was conducted in accordance with the Code of Ethics of the World Medical Association (Declaration of Helsinki) for experiments involving humans. The manuscript is in line with the Recommendations for the Conduct, Reporting, Editing and Publication of Scholarly Work in Medical Journals and aims for the inclusion of representative human populations (sex, age, and ethnicity) as per those recommendations. All procedures were performed in compliance with relevant laws and institutional guidelines and were approved by the Research Ethics Committee of the Hospital Universitario y Politécnico La Fe de Valencia (date: 21 September 2022; registration number: 2022-663-1). This study adhered to European and Spanish laws concerning personal data protection.

### 2.1. Participants and Study Design

A prospective single center (Hospital La Fe, Valencia, Spain) observational longitudinal cohort study that assessed pain relief outcomes after treatment with SCS using the WaveWriter Alpha™ device in patients diagnosed with small fiber PN, through formally established clinical criteria including intra-epidermal nerve fiber density (IENFD). Per established hospital protocol, patients were treated with SCS if they had not previously responded or were refractory to conventional pharmacological treatment and physical therapy. Implantation with a SCS device includes a routine 12-month-long clinical follow up protocol with assessments at baseline before intervention and post-implantation at 2, 4, 6, and 12 months post-activation.

Apart from having being treated with the WaveWriter Alpha™ device and having completed the 12-month follow-up protocol, additional inclusion criteria included: (i) age over 18 years old; (ii) the clinical diagnosis of small fiber polyneuropathy over a 1 year for DPN and CIAP or 2 years for CIPN; (iii) refractory to pharmacological treatment and for 1 year including GABAp; (v) morphine dose or equivalent < 120 mg/day; (vi) VAS > 5; (vii) BMI < 45; (viii) HbA1c < 10 (for DPN); and (ix) have not previously undergone spinal cord stimulation (for another pathology).

Exclusion criteria included: (i) documented history of substance abuse (narcotics, alcohol, etc.) or substance dependence during the past 6 months; (ii) systemic infection; (iii) pregnancy; (iv) participation in other clinical research with an active treatment group; (v) severe neurological or psychiatric pathology; (vi) current treatment with anticoagulant therapy; (vii) active skin infection in the access area of the technique; (vii) active oncological pathology; and (viii) current treatment with chemotherapy.

The study aimed to recruit patients with multiple etiologies: DPN, CIAP, CIPN, and other PNs (connective tissue diseases, sarcoidosis, vitamin B12 deficiency, monoclonal gammopathy, thyroid dysfunction, human immunodeficiency virus (HIV) infection, or alcohol or drug toxicity). Diagnosis was based on established clinical criteria.

The number of patients eligible was expected to be small as we were first limited by the total number of patients treated with SCS using the WaveWriter Alpha™ in the participating hospital. The device was only approved by the European Commission in late 2020 and it was not immediately available in Spain. Therefore, it has not been in use for very long, and the additional constraints that were placed on patient selection were expected to further lower the number of potential participants in the study.

Before inclusion in the study, patients had to have confirmed that they understood the information about the study and expressed their willingness to participate in it and meet all the requirements of the study by signing the corresponding informed consent form.

### 2.2. Spinal Cord Stimulation

**Figure 1:**
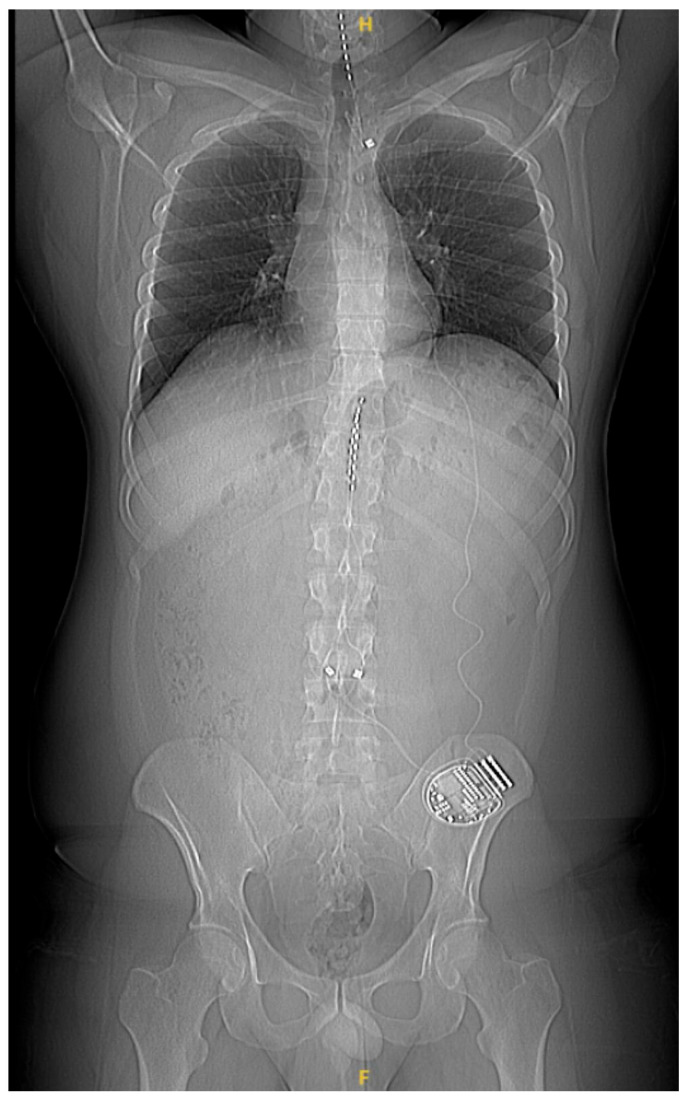

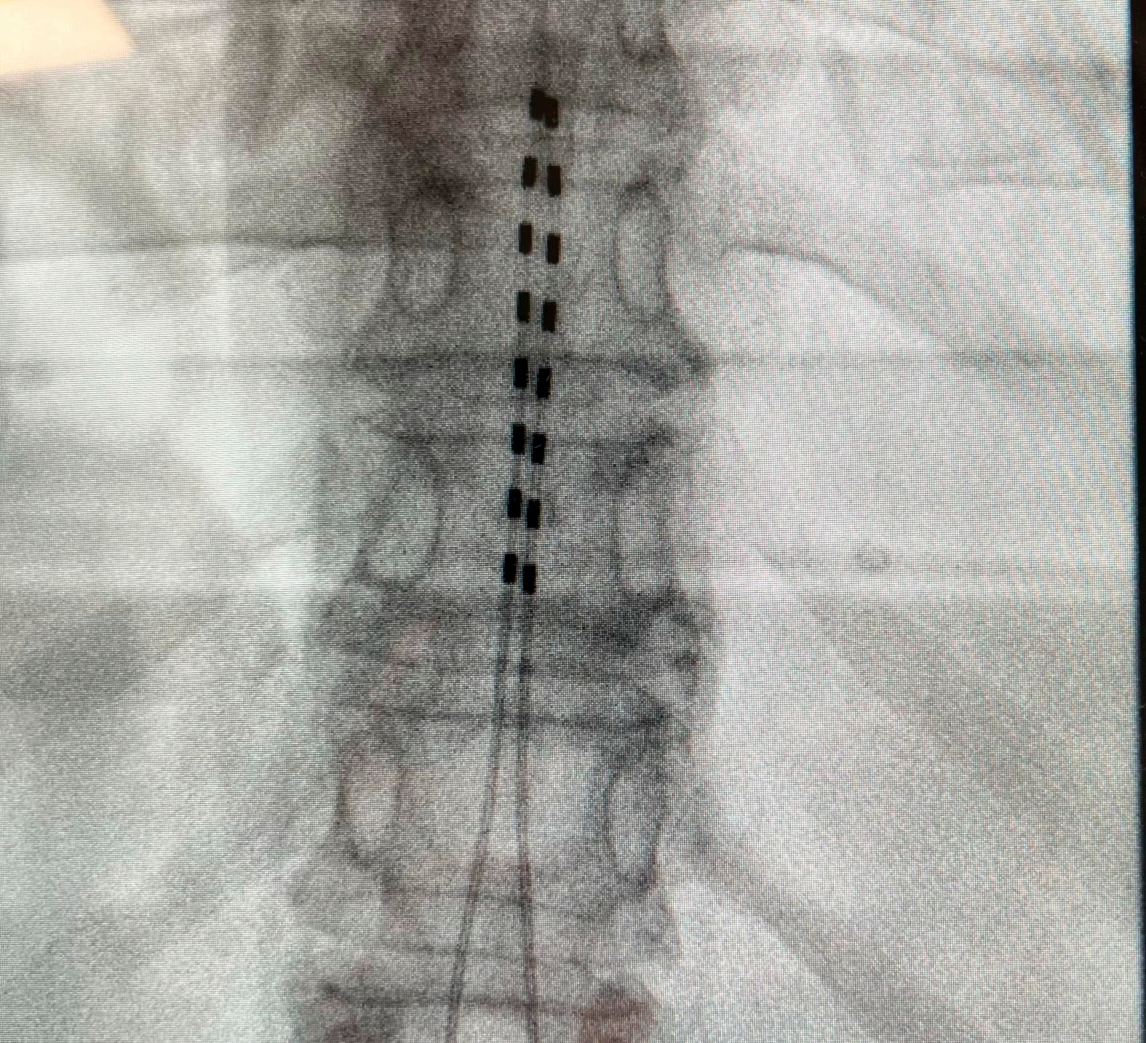
Typical lead placement.

Patients were implanted with epidural percutaneous leads targeting the lower extremities (T10–T11), and upper extremities (C4–C7) if necessary, and with an implantable pulse generator (IPG) system (Alpha WaveWriter^TM^, Boston Scientific Corporation, Valencia, CA, USA) (Figure 1). The implantation procedure and criteria for permanent implants were based on established standards and were performed in accordance with the center’s standard practice (pain relief ≥ 50% based on a trial phase).

SCS programming was individualized to each subject based on their personal preferences regarding the presence of SCS-induced paresthesia sensations (super-perception) or their absence (sub-perception). The SCS modalities chosen were the options currently offered by the device manufacturer for each category. (i) Super-perception: a combination of conventional paresthesia-based and a sub-perception (Contour© electric field at 50 Hz frequency, ~300 µs pulse-width, and amplitude ~40% of perception threshold), termed “Combination Therapy”; (ii) sub-perception: 90 Hz frequency, ~250 µs pulse-width, and amplitude ~40% of perception threshold, termed “Fast-Acting Sub-perception Therapy (FAST)”.

### 2.3. Clinical Assessments

Demographic information, medical history, and stimulation parameters, were collected for all patients, as documented as part of their routine clinical follow up. Clinical assessments were performed at baseline and post-implant (2, 4, 6 and 12 months post-activation) by certified physicians: a pain doctor, a neurologist, and rehabilitator. Outcomes studied include average pain intensity according to the visual analog scale (VAS), and the Douleur Neuropathique-4 (DN4) questionnaire; neuropathic symptoms according to the Neuropathic Pain Symptoms Inventory (NPSI) [23] and the short-form McGill Pain Questionnaire 2 (SF-MPQ-2) [24]; autonomic symptoms according to the SFN Symptoms Inventory Questionnaire (SFN-SIQ) [25] and the Survey for Autonomic Symptoms (SAS) [26]; sensory and small fiber nerve injury identified through a sensorimotor physical exam using the Utah Early Neuropathy Scale (UENS); functionality, assessed using the Global Assessment of Functioning (GAF) [27]; sleep, using the Chronic Pain Sleep Inventory Sleep Problem Index (CPSI-SPI) [28], the Clinician Global Impression of Change (CGI) scale, and the Patient Global Impression of Change (PGI) scale [29]; and quality of life, according to the 5-level EuroQol 5 Dimension system (EQ-5D-5L), with both its components assessed using the EQ-5D descriptive system and the EQ visual analog scale (EQ VAS) [30,31].

**Figure 2:**
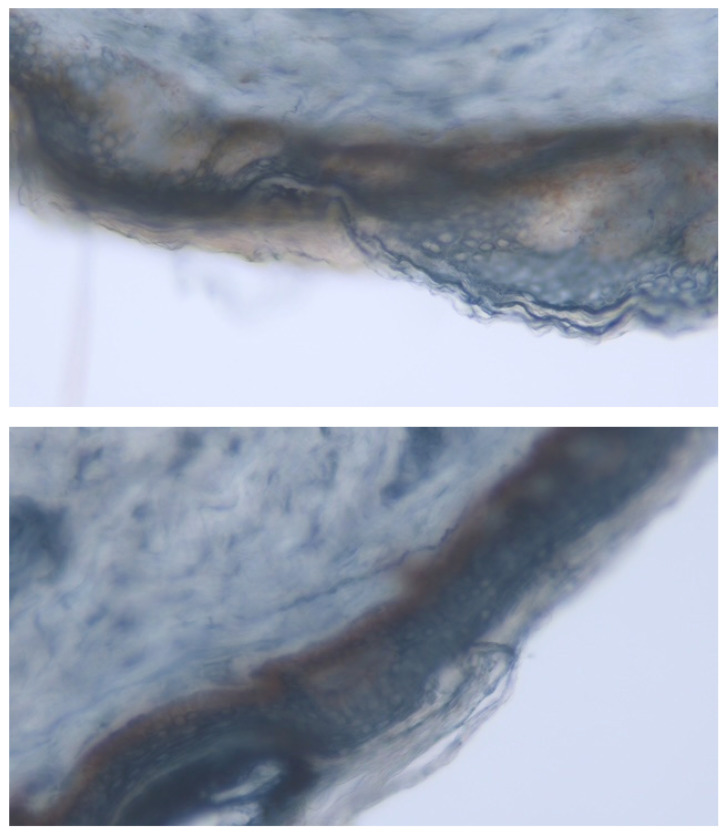
Intra-epidermal nerve fiber density via skin biopsy. The fibers are seen in a darker blue color, and those that go through or contact the outermost part of the skin are shown in a blue or brown color. As the cuts are thick (50 microns), it is often difficult to take pictures with the fiber in continuity.

Lastly, IENFD was performed at baseline for diagnostics purposes and at 12 months post-SCS to assess potential small fiber re-growth (Figure 2). The neurological hallmark of PNs includes preferential damage to the small nerve fibers at both somatic and autonomic divisions and the gold standard diagnosis of small fibers involvement requires the quantification of IENFD via skin biopsy and/or deficit in temperature threshold testing.

The smallest difference in outcome that was considered significant was the minimal clinically important difference (MCID), established for each outcome measurement. The MCID is the smallest change considered important or notable by the patient. It is a patient-centered concept that captures the magnitude of change, which can be positive or negative, and the value patients place on this change [32,33,34,35]. MCID values vary depending on the patient, clinical context, and estimation method [36]; however, some outcome measures do have previously established and recognized MCIDs.

MCID was determined for all outcomes, when possible, based on the currently available literature. For outcome measures of chronic pain such as DN4 or VAS, a two-point (ten-point scale) or 30% improvement was considered the MCID [37]. Given its sensitivity and strong correlation with patient satisfaction [38], a change of 1 in PGI/CGI is generally accepted as the MCID [39,40,41]. MCID for GAF was established at 4 [42]. For musculoskeletal conditions, the suggested MCID for SF-MPQ-2 is 5 [43]. There are few data on the MCID for the different measures of sleep quality in chronic pain patients. However, one study using a 0–100 sleep quality scale like that of CPSI-SPI documented a MCID of 6 [44]. The MCID was 0.03 points for the categorical scale of the EQ-5D [45]. For EQ-VAS, no single value has been established as the MCID, with previous studies presenting values from 6 to 12 [46,47,48,49,50,51]. For this study, the EQ-VAS MCID threshold was established at 8 following the latest evidence available [52]. For NPSI and SFN-SIQ, a MCID was not included due to an lack of appropriate literature. For UENS, establishing a MCID may not even be possible as it only assesses allodynia as present or absent and may therefore not be sensitive enough to evaluate improvement. While patients may improve, the UENS cannot detect it unless allodynia fully disappears.

### 2.4. Statistical Analysis

All data were analyzed with IBM SPSS Statistics. Analyses were performed for the sample as a whole and stratified by etiology. Subgroup analysis is a valuable tool to characterize treatment effect heterogeneity, and in this case, it was performed in order to study whether the response to treatment with SCS was different between the multiple etiologies studied (DPN, CIAP, and CIPN).

As part of the selection process, only patients with complete data, with the only exception being data related to IENFD, were included. The level of statistical significance was established in all cases at *p* < 0.05. The results of the analyses are expressed as frequency and percentage, mean ± standard deviation, and/or median and interquartile range.

Descriptive statistics were used for the demographic record (mean age and sex) and other medical variables (concomitant diseases). The normality of the data distribution was verified using the Shapiro–Wilk test. The paired Student’s *t*-test or the Mann–Whitney U test was used to determine the level of statistical significance of the results obtained.

## 3. Results

### 3.1. Participants

To date, 20 patients (DPN (*n* = 6), CIAP (*n* = 9), CIPN (*n* = 4), and other PNs (*n* = 1)) have been included in the study.

**Figure 3:**
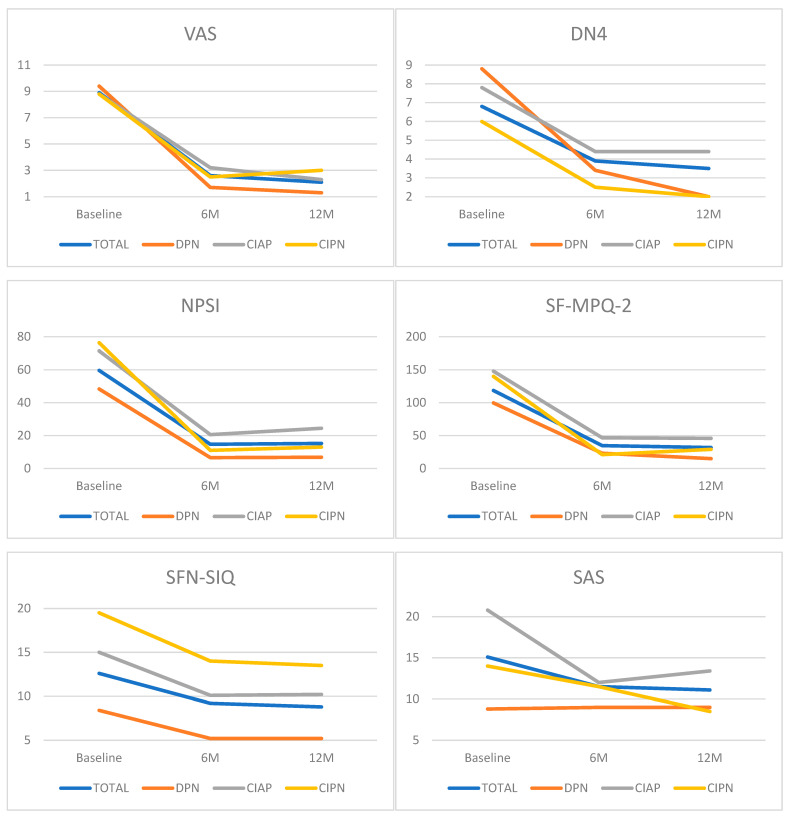

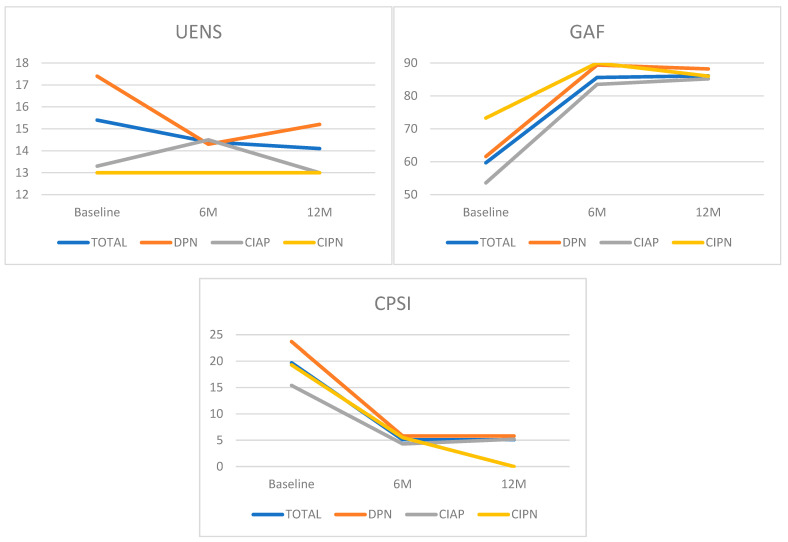

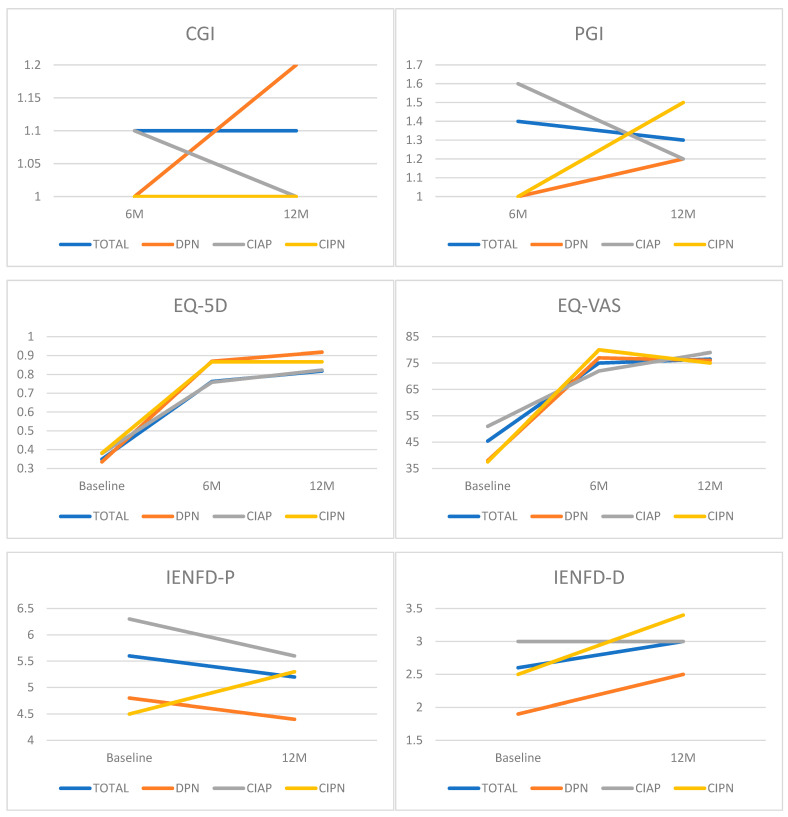
Clinical assessment outcome measurements at baseline and 6 and 12 months by etiology. For all outcomes, the point of measurement of the outcome is represented on the X-axis and the outcome score (points) is shown on the Y-axis. VAS: visual analog scale; DN4: Douleur Neuropathique-4; NPSI: Neuropathic Pain Symptom Inventory; SF-MPQ-2: short-form McGill Pain Questionnaire 2; SFN-SIQ: Small Fiber Neuropathy Symptom Inventory Questionnaire; SAS: Survey for Autonomic Symptoms; UENS: Utah Early Neuropathy Scale; GAF: Global Assessment of Functioning; CPSI-SPI: Chronic Pain Sleep Inventory Sleep Problem Index; CGI: Clinician Global Impression of Change; PGI: Patient Global Impression of Change; EQ-5D: five-level EuroQol Five-Dimension descriptive system; EQ VAS: five-level EuroQol Five-Dimension visual analog scale; IENFD-P: proximal intra-epidermal nerve fiber density; IENFD-D: distal intra-epidermal nerve fiber density. DPN: diabetic chronic polyneuropathy; CIAP: chronic idiopathic axonal polyneuropathy; CIPN: chemotherapy-induced chronic polyneuropathy.

All patients had a confirmed diagnosis of PN, and all met all the inclusion criteria and none of the exclusion criteria. Twelve patients (60%) had pain in the lower limbs only and, therefore, were implanted solely with dual thoracic leads, and eight patients (40%) had pain in both lower and upper limbs and, therefore, were implanted with dual thoracic leads and a cervical lead. One CIPN subject had to be excluded due to an atypical evolution (erythromelalgia, a torpid and very rapid progression that justifies therapeutic rescue with intravenous human nonspecific immunoglobulin) and, therefore, the current sample consists of 19 patients. General demographic and clinical characteristics are shown in Table 1. Figure 3 shows all outcome results throughout the follow-up period.

### 3.2. Baseline Assessments

#### 3.2.1. All Patients

Baseline assessments confirmed the severity of the referred patients (Table 2). Patients reported a high burden of pain intensity, neuropathic pain symptoms, and autonomic symptoms; had sensory and small fiber nerve injuries; and reported poor functionality, sleep, and quality of life. Patients presented marked decreased proximal and distal IENFDs at baseline.

#### 3.2.2. PN Subtypes

We also observed that PN etiology sub-groups differed in some clinical features at presentation (Table 2). For example, CIAP reported a higher burden of neuropathic symptoms, autonomic symptoms, and functionality. Meanwhile, CIPN patients reported worse neuropathic pain symptoms, autonomic symptoms, quality of life, and proximal IENFD. Lastly, DPN patients reported worse pain intensity, sensory and small fiber nerve injury, sleep, quality of life, and distal IENFD.

### 3.3. SCS Outcomes at Six Months

#### 3.3.1. All Patients

SCS was able to produce a significant and sustained (6 and 12 months) improvement in most of the clinical outcomes investigated across PN etiologies (N = 19) (Table 3). For those outcomes with an established MCID, all improvements observed were well above the established threshold.

At 6 months, subjects reported on average an improvement in pain intensity, neuropathic pain symptoms, autonomic symptoms, sensory and small fiber nerve injury, functionality, sleep, and quality of life. Also, both clinicians and patients had meaningful global impression of change at 6 months (CGI 1.1 and PGI 1.4).

#### 3.3.2. PN Subtypes

When analyses were segregated by etiology, we observed that, despite global results are similar across subtypes, there were some differences (Table 4, Table 5 and Table 6).

At 6 months post activation, the best outcome measurement scores for VAS were found in DPN patients, for DN4 in CIPN patients, for NPSI in DPN patients, for SF-MPQ-2 in CIPN patients, for SFN-SIQ in DPN patients, for SAS in DPN patients, for UENS in CIPN patients, for GAF in CIPN patients, for CPSI-SPI in CIAP patients, for CGI in DPN and CIPN patients, for PGI in DPN and CIPN patients, for EQ-5D in DPN patients, and for EQ-VAS in CIPN patients.

Improvements were observed for all outcome measures in all PN subtypes and for those outcomes with an established MCID, all improvements observed were well above the established threshold at six months.

In DPN patients, SCS was able to produce significant improvements in most outcome measurements at six months. Patients reported an improvement in pain intensity, neuropathic pain symptoms, autonomic symptoms, sensory and small fiber nerve injury, functionality, sleep, and quality of life. Also, both clinicians and patients recorded a meaningful global impression of change at 6 months (CGI 1.2 and PGI 1.2). A ~2% worsening was observed for the autonomic symptoms measurement (SAS).

In CIAP patients, SCS was able to produce a significant improvement in most outcome measurements at six months. Patients reported an improvement in pain intensity, neuropathic pain symptoms, autonomic symptoms, functionality, sleep, and quality of life. Also, both clinicians and patients recorded a meaningful global impression of change at 6 months (CGI 1.1 and PGI 1.6). A ~9% worsening was observed for sensory and small fiber nerve injury.

In CIPN patients, SCS was able to produce a significant improvement in most outcome measurements at six months. Patients reported an improvement in pain intensity, neuropathic pain symptoms, autonomic symptoms, functionality, sleep, and quality of life. Also, both clinicians and patients recorded a meaningful global impression of change at 6 months (CGI 1.0 and PGI 1.5). No change was observed for sensory and small fiber nerve injury.

### 3.4. SCS Outcomes at Twelve Months

#### 3.4.1. All Patients

At 12 months, subjects reported, on average, an improvement in pain intensity, neuropathic pain symptoms, autonomic symptoms, sensory and small fiber nerve injury, functionality, sleep, and quality of life. Also, both clinicians and patients recorded a meaningful global impression of change at 6 months (CGI 1.1 and PGI 1.3). IENFD-P decreased by ~18.2% from 5.6 to 5.2, while IENFD-D improved by 21.9% from 2.6 to 3.0.

#### 3.4.2. PN Subtypes

Improvements were observed for almost all outcome measures in all PN subtypes and for those outcomes with an established MCID, all improvements observed were well above the established threshold at twelve months.

At 12 months post activation, the best outcome measurement scores for VAS were found in DPN patients, for DN4 in DPN and CIPN patients, for NPSI in DPN patients, for SF-MPQ-2 in DPN patients, for SFN-SIQ in DPN patients, for SAS in CIPN patients, for UENS in CIAP and CIPN patients, for GAF in DPN patients, for CPSI-SPI in CIPN patients, for CGI in CIAP and CIPN patients, for PGI in DPN and CIAP patients, for EQ-5D in DPN patients, and for EQ-VAS in CIAP patients.

In DPN patients, at 12 months, SCS was able to produce a significant and sustained improvement in pain intensity, neuropathic pain symptoms, autonomic symptoms, sensory and small fiber nerve injury, functionality, sleep, and quality of life. Also, both clinicians and patients recorded a meaningful global impression of change at 6 months (CGI 1.2 and PGI 1.2). A ~2% worsening was observed for the autonomic symptoms measurement. IENFD-P decreased by ~12.9% from 4.8 to 4.4, while IENFD-D improved by 28.7% from 1.9 to 2.5.

In CIAP patients, at 12 months, SCS was able to produce a significant and sustained improvement in pain intensity, neuropathic pain symptoms, autonomic symptoms, sensory and small fiber nerve injury, functionality, sleep, and quality of life. Also, both clinicians and patients recorded a meaningful global impression of change at 6 months (CGI 1.0 and PGI 1.2). IENFD-P decreased by ~18.5% from 6.3 to 5.6, while IENFD-D improved by 0.5% from 3.0 to 3.0.

In CIPN patients, at 12 months, SCS was able to produce a significant and sustained improvement in pain intensity, neuropathic pain symptoms, autonomic symptoms, functionality, sleep, and quality of life. Also, both clinicians and patients recorded a meaningful global impression of change at 6 months (CGI 1.0 and PGI 1.5). No change was observed for sensory and small fiber nerve injury. IENFD-P improved by ~44.4% from 4.5 to 5.3 and IENFD-D improved by 21.9% from 2.5 to 3.4.

## 4. Discussion

Our current results support the use of SCS to effectively relieve pain, neuropathic symptoms, and autonomic symptoms and to improve functionality, sleep, and quality of life in patients with small fiber neuropathy, associated with diabetes or chemotherapy or with idiopathic origin, that is resistant to conventional pharmacological treatments. Outcomes were clinically meaningful, crossing MCID thresholds, and were maintained at 6 and 12 months after implantation, with both clinicians and patients recording a meaningful global impression of change. Distal IENFD improved at 12 months, while proximal IENFD decreased slightly.

The mechanism of action by which SCS alleviates pain in SFN involves a combination of neurochemical and neurophysiological effects that modulate pain perception and improve patient outcomes [53].

SCS can modulate nociceptive responses through the selective activation of nerve fibers and the enhancement of inhibitory pathways. High-frequency SCS primarily avoids paresthesia and targets smaller nerve fibers (such as A-delta and C fibers), while traditional low-frequency SCS predominantly activates large-diameter A-beta fibers. SCS alters the function of these fibers, which are involved in pain signaling, leading to reduced pain perception [54,55]. High-frequency and burst waveforms further refine this mechanism by exploiting fiber threshold accommodation, selectively blocking larger fibers while enabling smaller nociceptive fibers to transmit at reduced excitability levels. This differential activation modulates dorsal horn interneurons to release inhibitory neurotransmitters such as GABA, reducing neuronal excitability in nociceptive pathways and pain transmission [54,55]. This results in analgesia without the uncomfortable paresthesia common in low-frequency SCS [56].

Additionally, descending serotonergic pathways may enhance the analgesic effects by modulating the spinal processing of pain signals [57]. Increased levels of the neurotransmitters substance p and serotonin have been observed post-SCS, contributing to pain modulation [54]. The inhibition of SCS suppresses the release of excitatory neurotransmitters, such as excitatory amino acids, further dampening pain signals [54].

Emerging paradigms, such as burst and closed-loop systems, demonstrate potential in targeting both sensory-discriminative and emotional-affective components of neuropathic pain, offering improved patient outcomes for SFN [58]. The stimulation initiates a supraspinal–spinal feedback loop that enhances pain modulation through descending serotonergic pathways [55,59]. These innovations underscore the evolving understanding of SCS mechanisms tailored to treat SFN conditions effectively.

SCS has been shown to be a safe and useful tool for the management of a wide range of neurological disorders, such as the sequalae-related severe traumatic brain injury [60,61] or spasticity, a form of muscle hypertonia secondary to various diseases [62]. Furthermore, previous studies have already shown that SCS is effective in treating painful SFN, including DPN [16,20] and HIV-induced SFN [21].

Our study provides new data on topics that have not been fully addressed in the existing literature: (i) the use of SCS for CIAP; (ii) the use of cervical lead(s) is important to address the pain and neuropathic complaints of those patients who suffer from upper limb symptoms; (iii) some SCS modalities not previously tested are effective, including a low-frequency and energy-efficient sub-perception approach and a super-perception approach based on a combination of modalities (conventional + anatomical).

The data in this study provide additional evidence for some of these etiologies and new evidence for an idiopathic patient population, which is estimated to represent up to a third of (32%) chronic polyneuropathies [4]. In fact, CIAP were the largest subgroup in our cohort and included patients with possible channelopathies. The natural evolution and response to SCS can vary significantly in comparison with the other etiologies studied and also within a group due to its heterogeneity. This could be an explanation as to why the pain and neuropathic relief for this patient group was slightly more moderate.

The high burden of neuropathic pain symptoms in CIAP could not be explained by a longer duration of chronic pain, so it could be due to a more aggressive phenotype and progression of this umbrella etiology. Despite this higher severity, outcomes were highly satisfactory. This constitutes a tremendous benefit for these patients, many of whom had experienced pain, without alternative solutions, for many years. In any case, it is also worth mentioning the fact that our CIAP cases could represent a more severe profile than normal, since referral path was initiated for this study and some of the implanted patients had a long history of pain (>10 y) and medication failure.

The slightly more moderate analgesic response to SCS of CIAP vs. DPN could be simply due to the higher severity at presentation or to the fact that the mechanisms through which SCS exerts relief and recovery in DPN is different. One might speculate that pain and neurological symptom relief in DPN is mediated by the microvascular and nutrient recovery of the nerves, whereas for CIAP, other more complex mechanisms are involved, affecting the extent of nerve function recovery.

According to the results of the UENS sensorimotor physical exam, there was no significant recovery for sensory and small fiber nerve injury, despite the neuropathic pain symptom improvement. Only the allodynia/hyperesthesia section in UENS is specific to neuropathic symptoms, and it cannot capture a degree of change as it is scored in the binary as normal or present without regard to severity.

Both the proximal and distal IENFD values confirmed small fiber injury in all included patients at baseline. A previous study showed that in patients with a clinical diagnosis of neuropathy and abnormal IENFD, the mean distal to proximal ratio was 0.5 [63], which is in line with our results. In the assessment at 12 months post-SCS, distal IENFD improved while proximal IENFD decreased slightly. Recently, other authors have reported an IENFD increase in seven DPN patients after 24 months of dorsal root ganglion electrical stimulation [64]. Several factors could lead to this difference in results including methodology and duration which should be addressed in future research.

It was surprising to us to find that, even though length-dependent polyneuropathies end up affecting the distal parts of both lower and upper extremities, the use of epidural electrodes targeting both (i.e., cervical and thoracic) is not common practice in the literature. In this study, it was necessary for 40% of the patients. It seems that in previous studies, patients with upper limbs involvement were excluded or their symptoms were overlooked. In fact, the ability to implant more than two electrodes was a key factor in selecting a four-port SCS system. It is true, however, that progression in the extremities (symmetric vs. asymmetric) is dependent on the cause, but it was considered important to assess the degree of affection in upper limbs and, consequently, the consideration of use of lead(s) at the cervical level.

Regarding stimulation modality, it was found that both paresthesic and sub-paresthesic modalities were clinically valid and equally effective and that the choice was based solely on patient preference for SCS-induced tingling or not. The fact that many of these subjects suffered from pathological tingling due to their condition may explain why a higher percentage of patients (65%) preferred a sub-perception modality, although an even higher percentage was expected. It was preferred to use low electrical dosage to (i) avoid over-stimulation effects and (ii) minimize device re-charge burden. With the current settings, and also based on patient verbal reports, it is estimated that the average frequency of a battery recharge is once a week.

### 4.1. Clinical Implications

The clinical implications of the findings that SCS is effective in treating SFN are multifaceted and could significantly impact both individual patient care and broader healthcare practices. SFN often lacks effective treatments, particularly for patients who do not respond well to medications. SCS offers a non-pharmacological alternative for managing symptoms. Therefore, effective SCS therapy could decrease reliance on systemic medications, reducing the risk of side effects, drug tolerance, dependence, and interactions. This is particularly important for patients who require long-term symptom management. Neuropathic pain can significantly impair quality of life, limiting mobility, sleep, and daily activities. By providing targeted pain relief, SCS could improve physical function and emotional well-being, allowing patients to engage more fully in their lives. SCS can be tailored to the individual, with adjustable stimulation parameters to suit a patient’s specific pain profile. This personalized approach aligns with modern trends in medicine toward individualized treatment plans.

Although SCS implantation has a high upfront cost, it could reduce long-term healthcare expenditures by lowering medication use, hospital visits, and disability-related costs. Studies showing the durability of SCS’s effectiveness over years would reinforce its role as a mainstay treatment, encouraging its integration into standard chronic neuropathy care protocols.

### 4.2. Limitations

The primary limitation is the sample size. Hence, the current results should be interpreted as trends or observations rather than actual findings at this point. The aim is to reach a cohort of up to 40 cases. However, since the current data are satisfactory and some of the preliminary findings are noteworthy, they should be shared with the SCS community and the specialists involved in the care of peripheral neuropathy patients.

The longitudinal cohort study design presents several inherent challenges, especially due to its extended time frame. These challenges include the following: (i) the incomplete or interrupted follow-up of participants, leading to attrition and loss to follow-up over time; (ii) the difficulty in distinguishing the mutual influence between exposure and outcome; and (iii) the risk of drawing inaccurate conclusions if statistical methods do not account for the correlation between repeated measures within individuals [65].

The design of the study also includes the absence of a control group, which could be perceived as an important limitation. However, cohort studies do not always have a control group, because members of the cohort are primarily selected because they already share a particular characteristic. In this case, this characteristic was having received treatment with SCS, and in order to qualify for it, per hospital protocol, patients must not have previously responded or have been refractory to classical treatment methods. For adequate comparison with a control group, this group would have needed to share this specific characteristic and be made up by patients that are also already known to not respond to classical treatment methods but have not been treated with SCS. This group of patients would already be known to be unresponsive to classical treatment and therefore there would be no comparison between the effectiveness and safety of SCS with classical treatment. More importantly, it would also entail denying an available treatment option to long-suffering chronic patients, which would not be correct.

## 5. Conclusions

In conclusion, the findings indicate that spinal cord stimulation is an effective treatment for chronic polyneuropathies from multiple etiologies. SCS has the potential to be a meaningful treatment option for patients with painful SFN who are refractory to conventional drugs and therefore have limited alternatives, including those without a known cause (idiopathic). By providing an effective option for a debilitating condition, SCS represents a promising advance in neuromodulation therapies, offering hope to patients with limited alternatives while also challenging the healthcare system to integrate this technology effectively and equitably. It is essential that SFN specialists and pain practitioners carefully consider the adoption of SCS for diverse types of SFN.

Further research, including larger, randomized controlled trials to confirm findings, is necessary to determine how various SFN subtypes respond to treatment and the underlying mechanisms involved.

## Figures and Tables

**Table 1:** Demographic and clinical characteristics of the patients at baseline (*n* = 19).

Characteristic	*n* (%) or Mean (±SD) or Median (IQR)	Min.	Max.
**Age at first stage** (years)		33	80
Mean (±SD)	60.21 (±14.08)		
Median (IQR)	60.00 (51.00–71.00)		
**Sex**			
Female	14 (73.7%)	-	-
Male	5 (26.3%)	-	-
**IMC**		17.5	34.0
Mean (±SD)	25.61 (±4.72)		
Median (IQR)	26 (22.00–30.00)		
**Polyneuropathy type** N (%)			
Diabetic	6 (31.6%)	-	-
Idiopathic	9 (47.4%)	-	-
Chemotherapy-induced	3 (15.8%)	-	-
Other	1 (5.2%)	-	-
**Time of evolution** (years)		1	20
Mean (±SD)	8.34 (±6.01)		
Median (IQR)	5.50 (4.0–14.0)		

**Table 2:** Baseline assessment.

Outcomes			Total	DPN	CIAP	CIPN	*p*-Value *
**Pain**	**VAS**	Mean (±SD)	8.9 (±0.8)	9.4 (±0.7)	8.8 (±0.9)	8.8 (±0.76)	0.346
		Median (IQR)	9.0 (8.0–9.9)	9.5 (8.7–9.9)	8.0 (8.0–10.0)	8.5 (8.0–8.5)	-
	**DN4**	Mean (±SD)	6.8 (±1.5)	8.8 (±2.28)	7.8 (±0.8)	6.0 (±1.0)	0.025
		Median (IQR)	7.5 (5.5–8.7)	7.0 (4.5–9.0)	8.0 (7.0–8.5)	6.0 (5.0–6.0)	-
**Neuropathy**	**NPSI**	Mean (±SD)	59.6 (±23.0)	48.3 (±25.6)	71.4 (±13.5)	76.3 (±22.9)	0.019
		Median (IQR)	58.0 (39.7–77.5)	38.0 (25.5–75.0)	78.0 (58.0–81.5)	68.5 (50.0–68.0)	-
	**SF-MPQ-2**	Mean (±SD)	118.6 (±48.4)	99.6 (±56.4)	147.8 (±47.2)	140.0 (±28.9)	0.977
		Median (IQR)	104.5 (84.0–164.7)	102.0 (48.0–150.0)	165.0 (97.5–189.5)	134.0 (107.0–134.0)	-
**Autonomic**	**SFN-SIQ**	Mean (±SD)	12.6 (±5.9)	8.4 (±3.9)	15.0 (±6.4)	19.5 (±0.7)	0.999
		Median (IQR)	11.0 (8.2–17.7)	9.0 (5.5–11.0)	17.0 (8.5–20.5)	19.5 (19.0–19.5)	-
	**SAS**	Mean (±SD)	15.1 (±9.7)	8.8 (±3.0)	20.8 (±12.6)	14.0 (±1.4)	0.061
		Median (IQR)	13.0 (8.2–18.7)	8.0 (6.0–12.0)	19.0 (11.5–31.0)	14.0 (13.0–14.0)	-
**Physical exam**	**UENS**	Mean (±SD)	15.4 (±3.0)	17.4 (±2.7)	13.3 (±6.0)	13.0 (±4.2)	0.999
		Median (IQR)	16.0 (14.0–17.7)	18.0 (15.0–19.5)	14.0 (10.5–15.0)	13.0 (10.0–13.0)	-
**Functioning**	**GAF**	Mean (±SD)	59.7 (±10.7)	61.6 (±7.3)	53.6 (±12.6)	73.3 (±2.8)	0.993
		Median (IQR)	60.5 (51.0–70.0)	61.0 (55.5–68.0)	51.0 (45.5–63.0)	72.5 (70.0–72.5)	-
**Sleep**	**CPSI-SPI**	Mean (±SD)	19.7 (±9.3)	23.7 (±5.9)	15.4 (±12.5)	19.3 (±8.3)	0.360
		Median (IQR)	22.5 (14.25–26.0)	24.5 (19.0–28.0)	21.0 (2.5–25.5)	24.0 (22.0–24.0)	-
**Quality of life**	**EQ-5D**	Mean (±SD)	0.349 (±0.130)	0.335 (±0.126)	0.380 (±0.109)	0.382 (±0.155)	0.754
		Median (IQR)	0.422 (0.221–0.433)	0.416 (0.198–0.431)	0.433 (0.270–0.463)	0.382 (0.272–0.382)	-
	**EQ-VAS**	Mean (±SD)	45.4 (±18.6)	38.0 (±13.0)	51.0 (±24.1)	37.5 (±3.5)	0.322
		Median (IQR)	40.0 (30.0–55.0)	30.0 (30.0–50.0)	50.0 (32.5–70)	37.5 (35.0–37.5)	-
**Biopsy**	**IENFD Proximal**	Mean (±SD)	5.6 (±2.3)	4.8 (±2.3)	6.3 (±2.1)	4.5 (±3.5)	0.376
		Median (IQR)	6.6 (3.6–7.5)	4.4 (2.9–7.4)	6.7 (5.8–7.7)	4.4 (1.0–8.0)	-
	**IENFD Distal**	Mean (±SD)	2.6 (±1.9)	1.9 (±1.2)	3.0 (±2.3)	2.5 (±1.8)	0.607
		Median (IQR)	2.1 (1.0–3.6)	1.6 (1.4–2.4)	3.5 (0.5–4.6)	2.1 (1.0–4.5)	-

VAS: visual analog scale; DN4: Douleur Neuropathique-4; NPSI: Neuropathic Pain Symptom Inventory; SF-MPQ-2: short-form McGill Pain Questionnaire 2; SFN-SIQ: Small Fiber Neuropathy Symptom Inventory Questionnaire; SAS: Survey for Autonomic Symptoms; UENS: Utah Early Neuropathy Scale; GAF: Global Assessment of Functioning; CPSI-SPI: Chronic Pain Sleep Inventory Sleep Problem Index; CGI: Clinician Global Impression of Change; PGI: Patient Global Impression of Change; EQ-5D: five-level EuroQol Five-Dimension descriptive system; EQ VAS: five-level EuroQol Five-Dimension visual analog scale; IENFD: intra-epidermal nerve fiber density. * Comparison between DPN, CIAP, and CIPN was carried out using the Mann–Whitney U-test.

**Table 3:** Main outcomes for all patients (*n* = 19).

Outcomes			Baseline	2 Months	4 Months	6 Months	12 Months	Δ Baseline– 6 Months	Δ Baseline– 12 Months	*p*-Value ^a^
**Pain**	**VAS**	Mean (±SD)	8.9 (±0.8)	2.6 (±1.9)	2.7 (±1.7)	2.6 (±1.7)	2.1 (±1.6)	−6.3 (~71%)	−6.8 (~76%)	0.001
		Median (IQR)	9.0 (8.0–9.9)	3.0 (1.6–3.7)	3.0 (1.6–4.0	2.0 (1.0–3.5)	2.0 (0.7–3.5)	−7.0	−7.0	-
	**DN4**	Mean (±SD)	6.8 (±1.5)	4.1 (±2.4)	4.0 (±2.2)	3.9 (±2.1)	3.5 (±2.6)	−2.9 (~43%)	−3.3 (~49%)	0.001
		Median (IQR)	7.5 (5.5–8.7)	4.5 (3.2–7.5)	4.0 (3.0–6.7)	4.0 (2.0–5.5)	3.0 (1.0–6.0)	−3.5	−4.5	-
**Neuropathy**	**NPSI**	Mean (±SD)	59.6 (±23.0)	21.2 (±17.5)	20.1 (±18.4)	14.7 (±12.6)	15.2 (±16.7)	−44.9 (~75%)	−44.4 (~74%)	0.001
		Median (IQR)	58.0 (39.7–77.5)	28.0 (8.5–41.0)	18.5 (3.2–48.0)	13.0 (4.0–21.0)	7.0 (1.5–25.0)	−45.0	−51.0	-
	**SF-MPQ-2**	Mean (±SD)	118.6 (±48.4)	57.3 (±40.0)	54.8 (±41.3)	34.7 (±30.8)	31.8 (±34.0)	−83.9 (~71%)	−86.8 (~73%)	0.001
		Median (IQR)	104.5 (84.0–164.7)	56.0 (20.5–85.2)	37.0 (16.2–99.5)	19.0 (16.0–61.0)	18.0 (1.5–56.0)	−85.5	−86.5	-
**Autonomic**	**SFN-SIQ**	Mean (±SD)	12.6 (±5.9)	9.6 (±9.5)	9.7 (±5.7)	9.2 (±5.4)	8.8 (±5.3)	−3.4 (~27%)	−3.8 (~30%)	0.437
		Median (IQR)	11.0 (8.2–17.7)	9.5 (4.5–15.0)	8.5 (4.5–15.5)	10.0 (5.0–13.0)	7.0 (5.0–12.0)	−1.0	−4.0	-
	**SAS**	Mean (±SD)	15.1 (±9.7)	12.1 (±6.5)	12.1 (±6.5)	11.5 (±5.8)	11.1 (±5.7)	−3.6 (24%)	−4.0 (~26%)	0.831
		Median (IQR)	13.0 (8.2–18.7)	10.0 (7.0–19.7)	10.0 (7.0–19.7)	10.0 (8.0–15.5)	10.0 (7.0–14.0)	−3.0	−3.0	-
**Physical exam**	**UENS**	Mean (±SD)	15.4 (±3.0)	14.6 (±5.3)	14.4 (±4.5)	14.4 (±4.8)	14.1 (±2.9)	−1.0 (~6%)	−1.3 (~8%)	0.919
		Median (IQR)	16.0 (14.0–17.7)	14.0 (12.2–16.7)	14.0 (12.5–16.0)	14.0 (10.0–16.5)	14.0 (11.5–16.0)	−2.0	−2.0	-
**Functioning**	**GAF**	Mean (±SD)	59.7 (±10.7)	83.5 (±6.3)	85.0 (±6.0)	85.6 (±5.9)	86.1 (±6.1)	25.9 (~43%)	26.4 (~44%)	0.001
		Median (IQR)	60.5 (51.0–70.0)	85.0 (80.25–90.0)	87.5 (80.25–90.0)	87.0 (80.5–90.0)	90.0 (83.0–90.5)	26.5	29.5	-
**Sleep**	**CPSI-SPI**	Mean (±SD)	19.7 (±9.3)	6.8 (±8.2)	7.5 (±8.3)	5.1 (±6.6)	5.1 (±7.1)	−14.6 (~74%)	−14.6 (~74%)	0.001
		Median (IQR)	22.5 (14.25–26.0)	4.5 (0.0–11.7)	6.0 (0.0–12.7)	0.0 (0.0–11.0)	0.0 (0.0–11.0)	−22.5	−22.5	-
**Global impression**	**CGI**	Mean (±SD)	-	1.3 (±0.5)	1.1 (±0.3)	1.1 (±0.3)	1.1 (±0.4)	1.1	1.1	0.300
		Median (IQR)	-	1.0 (1.0–2.0)	1.0 (1.0–2.0)	1.0 (1.0–1.0)	1.0 (1.0–1.0)	1.0	1.0	-
	**PGI**	Mean (±SD)	-	1.6 (±0.5)	1.5 (±0.7)	1.4 (±0.6)	1.3 (±0.5)	1.4	1.3	0.395
		Median (IQR)	-	2.0 (1.0–2.0)	1.0 (1.0–2.0)	1.0 (1.0–2.0)	1.0 (1.0–2.0)	1.0	1.0	-
**Quality of life**	**EQ-5D**	Mean (±SD)	0.349 (±0.130)	0.735 (±0.227)	0.758 (±0.224)	0. 762 (±0.232)	0.818 (±0.247)	0.413 (~118%)	0.469 (~134%)	0.001
		Median (IQR)	0.422 (0.221–0.433)	0.775 (0.636–0.947)	0.790 (0.703–0.947)	0.790 (0.599–1.0)	1.0 (0.664–1.0)	0.368	0.578	-
	**EQ-VAS**	Mean (±SD)	45.4 (±18.6)	72.7 (±8.8)	71.5 (±10.5)	75.0 (±10.8)	76.5 (±10.7)	29.6 (~65%)	31.1 (~69%)	0.001
		Median (IQR)	40.0 (30.0–55.0)	70.0 (70.0–80.0)	70.0 (65.0 (80.0)	70.0 (67.5–85.0)	80.0 (70.0–87.5)	30.0	40.0	
**Biopsy**	**IENFD Proximal**	Mean (±SD)	5.6 (±2.3)	-	-	-	5.2 (±2.3) ^b^	-	18.2% (±58.7) ^c^	0.594
		Median (IQR)	6.6 (3.6–7.5)	-	-	-	4.7 (3.4–7.6) ^b^	-	5.2% (−14.9–35.4) ^c^	-
	**IENFD** **Distal**	Mean (±SD)	2.6 (±1.9)	-	-	-	3.0 (±1.2) ^b^	-	21.9% (±46.6) ^c^	0.442
		Median (IQR)	2.1 (1.0–3.6)	-	-	-	3.0 (1.9–4.2) ^b^	-	25.0% (−23.2–50.0) ^c^	-

VAS: visual analog scale; DN4: Douleur Neuropathique-4; NPSI: Neuropathic Pain Symptom Inventory; SF-MPQ-2: short-form McGill Pain Questionnaire 2; SFN-SIQ: Small Fiber Neuropathy Symptom Inventory Questionnaire; SAS: Survey for Autonomic Symptoms; UENS: Utah Early Neuropathy Scale; GAF: Global Assessment of Functioning; CPSI-SPI: Chronic Pain Sleep Inventory Sleep Problem Index; CGI: Clinician Global Impression of Change; PGI: Patient Global Impression of Change; EQ-5D: five-level EuroQol Five-Dimension descriptive system; EQ VAS: five-level EuroQol Five-Dimension visual analog scale; IENFD: intra-epidermal nerve fiber density. ^a^ Comparison between baseline, 2 months, 4 months, 6 months, and 12 months using Student’s *t*-test or Wilcoxon’s test. ^b^ *n* = 12. ^c^ Percentage of change calculated only for the *n* = 12 patients with data at baseline and at 12 months using: $\%\;change=\frac{\left(12\;months-Baseline\right)}{Baseline}\times 100.$

**Table 4:** Main outcomes for DPN (*n* = 6).

Outcomes			Baseline	2 Months	4 Months	6 Months	12 Months	Δ Baseline– 6 Months	Δ Baseline– 12 Months	*p*-Value ^a^
**Pain**	**VAS**	Mean (±SD)	9.4 (±0.7)	1.9 (±0.7)	2.1 (±1.1)	1.7 (±0.8)	1.3 (±1.1)	−7.7 (~82%)	−8.1 (~86%)	0.001
		Median (IQR)	9.5 (8.7–9.9)	2.0 (1.2–2.5)	2.0 (1.2–3.0)	1.5 (1.0–2.5)	1.0 (0.5–2.2)	−8.0	−8.5	-
	**DN4**	Mean (±SD)	8.8 (±2.28)	3.6 (±1.1)	3.4 (±1.7)	3.4 (±2.1)	2.0 (±2.1)	−5.4 (~61%)	−6.8 (~77%)	0.001
		Median (IQR)	7.0 (4.5–9.0)	4.0 (2.5–4.5)	3.0 (2.0–5.0)	3.0 (1.5–5.5)	2.0 (1.0–4.5)	−4.0	−5.0	-
**Neuropathy**	**NPSI**	Mean (±SD)	48.3 (±25.6)	16.0 (±17.8)	14.8 (±20.8)	6.6 (±9.9)	6.8 (±10.9)	−41.7 (~86%)	−41.5 (86~%)	0.002
		Median (IQR)	38.0 (25.5–75.0)	5.0 (2.5–35.0)	4.0 (2.5–32.5)	2.0 (0.5–15.0)	3.0 (0.0–15.5)	−36.0	−35.0	-
	**SF-MPQ-2**	Mean (±SD)	99.6 (±56.4)	37.4 (±39.8)	35.4 (±41.3)	23.0 (±22.2)	15.0 (±25.5)	−76.6 (~77%)	−84.6 (~85%)	0.004
		Median (IQR)	102.0 (48.0–150.0)	16.0 (5.0–80.0)	16.0 (9.0–71.5)	18.0 (8.0–40.5)	5.0 (0.0–35.0)	−84.0	−97.0	-
**Autonomic**	**SFN-SIQ**	Mean (±SD)	8.4 (±3.9)	5.4 (±3.4)	5.4 (±3.4)	5.2 (±3.8)	5.2 (±3.8)	−3.2 (~38%)	−3.2 (~38%)	0.575
		Median (IQR)	9.0 (5.5–11.0)	4.0 (3.0–8.5)	4.0 (3.0–8.5)	4.0 (2.0–9.0)	4.0 (2.0–9.0)	−5.0	−5.0	-
	**SAS**	Mean (±SD)	8.8 (±3.0)	8.0 (±3.1)	8.0 (±3.2)	9.0 (±3.3)	9.0 (±3.3)	0.2 (~2%)	0.2 (~2%)	0.987
		Median (IQR)	8.0 (6.0–12.0)	8.0 (5.0–11.0)	8.0 (5.0–11.0)	9.0 (6.0–12.0)	9.0 (7.0–21.5)	1.0	1.0	-
**Physical exam**	**UENS**	Mean (±SD)	17.4 (±2.7)	16.2 (±3.5)	15.6 (±2.7)	14.3 (±3.9)	15.2 (±3.1)	−3.1 (~18%)	−2.2 (~13%)	0.520
		Median (IQR)	18.0 (15.0–19.5)	16.0 (13.0–19.5)	16.0 (13.0–18.0)	16.0 (10.0–18.0)	16.0 (10.0–18.0)	−2.0	−2.0	-
**Functioning**	**GAF**	Mean (±SD)	61.6 (±7.3)	87.2 (±4.1)	89.4 (±1.3)	89.4 (±2.5)	88.2 (±2.9)	27.8 (~45%)	26.6 (~43%)	0.001
		Median (IQR)	61.0 (55.5–68.0)	90.0 (83.0–90.0)	90.0 (88.5–90.0)	90.0 (87.5–91.0)	90.0 (85.0–90.5)	29.0	29.0	-
**Sleep**	**CPSI-SPI**	Mean (±SD)	23.7 (±5.9)	6.7 (±6.3)	6.2 (±8.0)	5.8 (±8.3)	5.8 (±8.3)	−17.9 (~76%)	−17.9 (~76%)	0.001
		Median (IQR)	24.5 (19.0–28.0)	9.0 (0.0–12.5)	2.0 (0.0–18.0)	0.0 (0.0–14.5)	0.0 (0.0–14.5)	−24.5	−24.5	-
**Global impression**	**CGI**	Mean (±SD)	-	1.2 (±0.4)	1.2 (±0.4)	1.0 (±0.0)	1.2 (±0.4)	1.0	1.2	0.771
		Median (IQR)	-	1.0 (1.0–1.5)	1.0 (1.0–1.5)	1.0 (1.0–1.0)	1.0 (1.0–1.5)	1.0	1.0	-
	**PGI**	Mean (±SD)	-	1.6 (±0.5)	1.2 (±0.5)	1.0 (±0.0)	1.2 (±0.4)	1.0	1.2	0.164
		Median (IQR)	-	2.0 (1.0–2.0)	1.0 (1.0–1.5)	1.0 (1.0–1.0)	1.0 (1.0–1.5)	1.0	1.0	-
**Quality of life**	**EQ-5D**	Mean (±SD)	0.335 (±0.126)	0.855 (±0.197)	0.904 (±0.131)	0.870 (±0.186)	0.918 (±0.181)	0.535 (~160%)	0.583 (~174%)	0.001
		Median (IQR)	0.416 (0.198–0.431)	1.0 (0.639–1.0)	1.0 (0.760–1.0)	1.0 (0.677–1.0)	1.0 (0.797–1.0)	0.584	0.584	-
	**EQ-VAS**	Mean (±SD)	38.0 (±13.0)	74.0 (±5.47)	72.0 (±13.0)	77.0 (±9.74)	76.0 (±11.4)	39.0 (~103%)	38.0 (~100%)	0.001
		Median (IQR)	30.0 (30.0–50.0)	70.0 (70.0–80.0)	80.0 (60.0–80.0)	80.0 (67.5–85)	80.0 (65.0–85.0)	50.0	50.0	-
**Biopsy**	**IENFD Proximal**	Mean (±SD)	4.8 (±2.3)	-	-	-	4.4 (±2.3) ^b^	-	12.9 (±21.8) ^c^	0.767
		Median (IQR)	4.4 (2.9–7.4)	-	-	-	3.4 (3.1–6.7) ^b^	-	8.5 (−8.3–35.4) ^c^	-
	**IENFD** **Distal**	Mean (±SD)	1.9 (±1.2)	-	-	-	2.5 (±0.6) ^b^	-	28.7 (±49.1) ^c^	0.291
		Median (IQR)	1.6 (1.4–2.4)	-	-	-	2.5 (1.9–3.0) ^b^	-	30.0 (−17.6–74.0) ^c^	-

VAS: visual analog scale; DN4: Douleur Neuropathique-4; NPSI: Neuropathic Pain Symptoms Inventory; SF-MPQ-2: short-form McGill Pain Questionnaire 2; SFN-SIQ: Small Fiber Neuropathy Symptom Inventory Questionnaire; SAS: Survey for Autonomic Symptoms; UENS: Utah Early Neuropathy Scale; GAF: Global Assessment of Functioning; CPSI-SPI: Chronic Pain Sleep Inventory Sleep Problem Index; CGI: Clinician Global Impression of Change; PGI: Patient Global Impression of Change; EQ-5D: five-level EuroQol Five-Dimension descriptive system; EQ VAS: five-level EuroQol Five-Dimension visual analog scale; IENFD: intra-epidermal nerve fiber density. ^a^ Comparison between baseline, 2 months, 4 months, 6 months, and 12 months using Student’s *t*-test or Wilcoxon’s test. ^b^ *n* = 4. ^c^ Percentage of change calculated only for the *n* = 4 patients with data at baseline and at 12 months using: $\%\;change=\frac{\left(12\;months-Baseline\right)}{Baseline}\times 100.$

**Table 5:** Main outcomes for CIAP (*n* = 9).

Outcomes			Baseline	2 Months	4 Months	6 Months	12 Months	Δ Baseline– 6 Months	Δ Baseline– 12 Months	*p*-Value ^a^
**Pain**	**VAS**	Mean (±SD)	8.8 (±0.9)	3.4 (±2.2)	3.4 (±1.9)	3.2 (±2.1)	2.3 (±2.04)	−5.6 (~64%)	−6.5 (~74%)	0.001
		Median (IQR)	8.0 (8.0–10.0)	3.0 (1.2–2.5)	3.0 (2.0–4.5)	3.2 (1.2–4.6)	3.0 (0.5–2.2)	−4.8	−5.0	-
	**DN4**	Mean (±SD)	7.8 (±0.8)	6.2 (±2.4)	5.4 (±2.5)	4.4 (±1.9)	4.4 (±3.0)	−3.4 (~44%)	−3.4 (~44%)	0.027
		Median (IQR)	8.0 (7.0–8.5)	6.0 (4.0–8.5)	5.0 (3.0–8.0)	4.0 (3.0–5.7)	5.0 (1.5–7.0)	−4.0	−3.0	-
**Neuropathy**	**NPSI**	Mean (±SD)	71.4 (±13.5)	38.6 (±15.3)	33.6 (±22.1)	20.6 (±14.5)	24.4 (±22.5)	−50.8 (~71%)	−47.0 (~66%)	0.001
		Median (IQR)	78.0 (58.0–81.5)	41.0 (23.0–53.0)	45.0 (10.5–51.0)	17.0 (9.5–30.5)	24.0 (2.0–47.0)	−61.0	−54.0	-
	**SF-MPQ-2**	Mean (±SD)	147.8 (±47.2)	76.4 (±43.0)	75.8 (±49.7)	46.6 (±37.6)	45.8 (±45.5)	−101.2 (~68%)	−102.0 (~69%)	0.002
		Median (IQR)	165.0 (97.5–189.5)	76.0 (35.5–117.5)	80.0 (27.0–122.5)	40.5 (16.0–77.5)	46.0 (1.5–90.0)	−124.5	−119.0	-
**Autonomic**	**SFN-SIQ**	Mean (±SD)	15.0 (±6.4)	11.8 (±4.4)	12.2 (±6.5)	10.1 (±6.2)	10.2 (±6.4)	−4.9 (~33%)	−4.8 (~32%)	0.506
		Median (IQR)	17.0 (8.5–20.5)	10.0 (8.0–16.5)	10.0 (6.5–19.0)	8.5 (6.2–15.5)	7.0 (6.0–16.0)	−8.5	−10.0	-
	**SAS**	Mean (±SD)	20.8 (±12.6)	15.2 (±7.6)	15.2 (±7.6)	12.0 (±7.8)	13.4 (±7.5)	−8.8 (~42%)	−7.4 (~36%)	0.418
		Median (IQR)	19.0 (11.5–31.0)	19.0 (7.0–21.5)	19.0 (7.0–21.5)	9.5 (7.2–19.5)	10.0 (7.0 21.5)	−9.5	−9.0	-
**Physical exam**	**UENS**	Mean (±SD)	13.3 (±6.0)	13.0 (±6.3)	14.2 (±5.7)	14.5 (±6.0)	13.0 (±2.4)	1.2 (~9%)	−0.3 (~2%)	0.845
		Median (IQR)	14.0 (10.5–15.0)	13.0 (10.5–15.0)	14.0 (10.5–15.0)	14.0 (10.2–18.2)	14.0 (10.5–15.0)	0	0	-
**Functioning**	**GAF**	Mean (±SD)	53.6 (±12.6)	79.2 (±11.4)	82.2 (±5.6)	83.5 (±6.8)	85.2 (±8.8)	29.9 (~56%)	31.6 (~59%)	0.001
		Median (IQR)	51.0 (45.5–63.0)	81.0 (70.0–87.5)	81.0 (77.5–87.5)	86.0 (78.75–89.2)	90.0 (77.5–90.5)	35.0	39.0	-
**Sleep**	**CPSI-SPI**	Mean (±SD)	15.4 (±12.5)	7.2 (±0.0)	7.2 (±10.7)	4.3 (±6.3)	5.2 (±7.9)	−11.1 (~72%)	−10.2 (~66%)	0.060
		Median (IQR)	21.0 (2.5–25.5)	0.0 (0.0–18.0)	0.0 (0.0–18.0)	2.0 (0.0–7.7)	0.0 (0.0–13.0)	−21.0	−21.0	-
**Global impression**	**CGI**	Mean (±SD)	-	1.4 (±0.5)	1.4 (±0.5)	1.1 (±0.3)	1.0 (±0.0)	1.1	1.0	0.243
		Median (IQR)	-	1.0 (1.0–2.0)	1.0 (1.0–2.0)	1.0 (1.0–1.0)	1.0 (1.0–1.0)	1.0	1.0	-
	**PGI**	Mean (±SD)	-	1.6 (±0.5)	1.6 (±0.5)	1.6 (±0.7)	1.2 (±0.4)	1.6	1.2	0.173
		Median (IQR)	-	2.0 (1.0–2.0)	2.0 (1.0–2.0)	1.0 (1.0–2.0)	1.0 (1.0–1.5)	1.0	1.0	-
**Quality of life**	**EQ-5D**	Mean (±SD)	0.380 (±0.109)	0.713 (±0.094)	0.719 (±0.108)	0.758 (±0.191)	0.823 (±0.169)	0.378 (~99%)	0.443 (~117%)	0.001
		Median (IQR)	0.433 (0.270–0.463)	0.760 (0.760–1.0)	0.790 (0.614–0.790)	0.790 (0.579–0.947)	0.790 (0.664–1.0)	0.357	0.357	-
	**EQ-VAS**	Mean (±SD)	51.0 (±24.1)	70.0 (±12.2)	68.0 (±7.6)	72.0 (±13.1)	79.0 (±8.9)	21.0 (~41%)	28.0 (~55%)	0.001
		Median (IQR)	50.0 (32.5–70)	70 (60.0–80.0)	70.0 (60.0–75.0)	70.0 (60.0–75.0)	80.0 (70.0–87.5)	20.0	30.0	-
**Biopsy**	**IENFD Proximal**	Mean (±SD)	6.3 (±2.1)	-	-	-	5.6 (±2.0) ^b^	-	18.5 (±88.6) ^c^	0.477
		Median (IQR)	6.7 (5.8–7.7)	-	-	-	5.1 (3.9–7.6) ^b^	-	−15.0 (−36.2–89.9) ^c^	-
	**IENFD** **Distal**	Mean (±SD)	3.0 (±2.3)	-	-	-	3.0 (±1.5) ^b^	-	0.5 (±45.2) ^c^	0.999
		Median (IQR)	3.5 (0.5–4.6)	-	-	-	3.4 (1.5–4.4) ^b^	-	0.9 (−43.1–43.7) ^c^	-

VAS: visual analog scale; DN4: Douleur Neuropathique-4; NPSI: Neuropathic Pain Symptom Inventory; SF-MPQ-2: short-form McGill Pain Questionnaire 2; SFN-SIQ: Small Fiber Neuropathy Symptom Inventory Questionnaire; SAS: Survey for Autonomic Symptoms; UENS: Utah Early Neuropathy Scale; GAF: Global Assessment of Functioning; CPSI-SPI: Chronic Pain Sleep Inventory Sleep Problem Index; CGI: Clinician Global Impression of Change; PGI: Patient Global Impression of Change; EQ-5D: five-level EuroQol Five-Dimension descriptive system; EQ VAS: five-level EuroQol Five-Dimension visual analog scale; IENFD: intra-epidermal nerve fiber density. ^a^ Comparison between baseline, 2 months, 4 months, 6 months, and 12 months using Student’s *t*-test or Wilcoxon’s test. ^b^ *n* = 5. ^c^ Percentage of change calculated only for the *n* = 5 patients with data at baseline and at 12 months using: $\%\;change=\frac{\left(12\;months-Baseline\right)}{Baseline}\times 100.$

**Table 6:** Main outcomes for CIPN (*n* = 3).

Outcomes			Baseline	2 Months	4 Months	6 Months	12 Months	Δ Baseline– 6 Months	Δ Baseline– 12 Months	*p*-Value ^a^
**Pain**	**VAS**	Mean (±SD)	8.8 (±0.76)	2.0 (±1.0)	2.3 (±0.6)	2.5 (±0.7)	3.0 (±1.4)	−6.3 (~72%)	−5.8 (~66%)	0.001
		Median (IQR)	8.5 (8.0–8.5)	2.5 (2.0–2.5)	2.5 (2.0–2.5)	2.5 (2.0–1.0)	3.0 (2.0–3.0)	−6.0	−5.5	-
	**DN4**	Mean (±SD)	6.0 (±1.0)	2.0 (±1.7)	2.0 (±1.7)	2.5 (±2.1)	2.0 (±1.4)	−3.5 (~58%)	−4.0 (~67%)	0.038
		Median (IQR)	6.0 (5.0–6.0)	2.5 (1.0–2.5)	2.5 (1.0–2.5)	2.5 (1.0–2.5)	2.0 (1.0–2.0)	−3.5	−4.0	-
**Neuropathy**	**NPSI**	Mean (±SD)	76.3 (±22.9)	11.0 (±9.5)	10.3 (±7.1)	11.0 (±2.8)	13.0 (±8.5)	−65.3 (~86%)	−63.3 (~83%)	0.999
		Median (IQR)	68.5 (50.0–68.0)	14.0 (6.0–14.0)	13.5 (9.0–13.5)	11.0 (9.0–11.0)	13.0 (7.0–13.0)	−57.5	−55.5	-
	**SF-MPQ-2**	Mean (±SD)	140.0 (±28.9)	17.3 (±16.1)	25.3 (±9.7)	21.0 (±5.6)	29.0 (±15.5)	−119.0 (~85%)	−111.0 (~79%)	0.996
		Median (IQR)	134.0 (107.0–134.0)	22.0 (8.0–22.0)	29.5 (23.0–29.5)	21.0 (12.0–17.0)	29.0 (18.0–29.0)	−113.0	−111.0	-
**Autonomic**	**SFN-SIQ**	Mean (±SD)	19.5 (±0.7)	15.5 (±2.1)	15.5 (±2.1)	14.0 (±0.0)	13.5 (±0.7)	−5.5 (~28%)	−6.0 (~31%)	0.006
		Median (IQR)	19.5 (19.0–19.5)	15.5 (14.0–15.5)	14.0 (14.0–14.0)	14.0 (14.0–14.0)	13.5 (13.0–13.5)	−5.0	−6.0	-
	**SAS**	Mean (±SD)	14.0 (±1.4)	11.5 (±2.1)	11.5 (±2.1)	11.5 (±2.1)	8.5 (±6.4)	−2.5 (~18%)	−5.5 (~39%)	0.480
		Median (IQR)	14.0 (13.0–14.0)	11.5 (10.0–11.5)	11.5 (10.0–11.5)	11.5 (10.0–11.5)	8.5 (4.0–8.5)	−2.5	−5.5	-
**Physical exam**	**UENS**	Mean (±SD)	13.0 (±4.2)	12.0 (±2.8)	12.0 (±2.8)	13.0 (±4.2)	13.0 (±4.2)	0 (~0%)	0 (~0%)	0.991
		Median (IQR)	13.0 (10.0–13.0)	12.0 (10.0–12.0)	12.0 (10.0–12.0)	13.0 (10.0–13.0)	13.0 (10.0–13.0)	0	0	-
**Functioning**	**GAF**	Mean (±SD)	73.3 (±2.8)	88.3 (±2.9)	86.3 (±2.9)	90.0 (±0.1)	86.0 (±7.1)	16.7 (~23%)	12.7 (~17%)	0.001
		Median (IQR)	72.5 (70.0–72.5)	87.5 (85.0–87.5)	88.0 (88.0–88.0)	90.0 (90.0–90.0)	86.0 (81.0–86.0)	17.5	13.5	-
**Sleep**	**CPSI**	Mean (±SD)	19.3 (±8.3)	0.0 (±0.0)	5.0 (±5.0)	5.5 (±7.7)	0.0 (±0.0)	−13.8 (~72%)	−19.3 (~100%)	-
		Median (IQR)	24.0 (22.0–24.0)	5.0 (0.0–5.5)	5.0 (0.0–5.5)	5.5 (0.0–5.5)	0.0 (±0.0)	−18.5	−24.0	-
**Global impression**	**CGI**	Mean (±SD)	-	1.0 (±0.0)	1.0 (±0.0)	1.0 (±0.0)	1.0 (±0.0)	1.0	1.0	1.0
		Median (IQR)	-	1.0 (1.0–1.0)	1.0 (1.0–1.0)	1.0 (1.0–1.0)	1.0 (1.0–1.0)	1.0	1.0	-
	**PGI**	Mean (±SD)	-	1.0 (±0.0)	1.0 (±0.0)	1.0 (±0.0)	1.5 (±0.7)	1.0	1.5	1.0
		Median (IQR)	-	1.0 (1.0–1.0)	1.0 (1.0–1.0)	1.0 (1.0–1.0)	1.5 (1.0–1.5)	1.0	1.5	-
**Quality of life**	**EQ-5D**	Mean (±SD)	0.382 (±0.155)	0.895 (±0.148)	0.895 (±0.148)	0.867 (±0.187)	0.867 (±0.187)	0.485 (~127%)	0.485 (~127%)	0.005
		Median (IQR)	0.382 (0.272–0.382)	0.895 (0.790–0.895)	0.895 (0.790–0.895)	0.867 (0.735–0.867)	0.867 (0.735–0.867)	0.485	0.485	-
	**EQ-VAS**	Mean (±SD)	37.5 (±3.5)	77.5 (±10.6)	80.0 (±14.1)	80.0 (±14.1)	75.0 (±21.2)	42.5 (~113%)	37.5 (~100%)	0.006
		Median (IQR)	37.5 (35.0–37.5)	77.5 (70.0–75.5)	80.0 (70.0–80.0)	80.0 (70.0–80.0)	75.0 (60.0–75.0)	42.5	37.5	-
**Biopsy**	**IENFD Proximal**	Mean (±SD)	4.5 (±3.5)	-	-	-	5.3 (±4.9) ^b^	-	44.4 (±50.4) ^c^	0.823
		Median (IQR)	4.4 (1.0–8.0)	-	-	-	5.3 (1.8–8.7) ^b^	-	44.4 (8.75–80.0) ^c^	-
	**IENFD** **Distal**	Mean (±SD)	2.5 (±1.8)	-	-	-	3.4 (±2.0) ^b^	-	53.3 (±66.0) ^c^	0.579
		Median (IQR)	2.1 (1.0–4.5)	-	-	-	3.4 (2.0–4.8) ^b^	-	53.3 (6.67–100.0) ^c^	-

VAS: visual analog scale; DN4: Douleur Neuropathique-4; NPSI: Neuropathic Pain Symptom Inventory; SF-MPQ-2: short-form McGill Pain Questionnaire 2; SFN-SIQ: Small Fiber Neuropathy Symptom Inventory Questionnaire; SAS: Survey for Autonomic Symptoms; UENS: Utah Early Neuropathy Scale; GAF: Global Assessment of Functioning; CPSI-SPI: Chronic Pain Sleep Inventory Sleep Problem Index; CGI: Clinician Global Impression of Change; PGI: Patient Global Impression of Change; EQ-5D: five-level EuroQol Five-Dimension descriptive system; EQ VAS: five-level EuroQol Five-Dimension visual analog scale; IENFD: intra-epidermal nerve fiber density. ^a^ Comparison between baseline, 2 months, 4 months, 6 months, and 12 months using Student’s *t*-test or Wilcoxon’s test. ^b^ *n* = 2. ^c^ Percentage of change calculated only for the *n* = 2 patients with data at baseline and at 12 months using: $\%\;change=\frac{\left(12\;months-Baseline\right)}{Baseline}\times 100.$

## Data Availability

The datasets presented in this article are not readily available because of patient privacy limitations.
